# Prioritizing indigenous health equity in health registers: an environmental scan of strategies for equitable ascertainment and quality data

**DOI:** 10.1186/s41256-022-00250-6

**Published:** 2022-07-19

**Authors:** Karen Wright, Aria Dehar, N. Susan Stott, Anna Mackey, Alexandra Sorhage, Rachel Tapera, Sîan A. Williams

**Affiliations:** 1 Faculty of Medical and Health Sciences, Te Kupenga Hauora Māori, Auckland, New Zealand; 2 Department of Surgery, University of Auckland, Auckland, New Zealand; 3 New Zealand Cerebral Palsy Register, Starship Child Health, Auckland, New Zealand; 4 Liggins Institute, University of Auckland, Auckland, New Zealand; 5 Curtin School of Allied Health, Curtin University, Perth, Australia

**Keywords:** Indigenous health, Health equity, Health register, Kaupapa Māori, Ascertainment, Data quality, Cerebral palsy

## Abstract

**Background:**

Cerebral palsy (CP) registers serve as instrumental tools to support development of care pathways, preventative strategies, and health gains. Such health gains, however, are not always universal, with Indigenous health inequities common. To support Indigenous health, health registers need complete, consistent, and high-quality data. The aim of this study was to identify perceived barriers to the ascertainment of Indigenous peoples on health registers and to collate strategies supporting comprehensive ascertainment and achievement of high-quality Indigenous data.

**Methods:**

Environmental scanning methods were utilized within a Kaupapa Māori theoretical framework, which aims to produce research that is transformational and supportive of Indigenous health gain. Knowledge and insights were obtained from CP registers in countries with Indigenous populations and complemented by information from health registers in Aotearoa New Zealand (NZ). Data collection methods included an online survey and scan of organizational websites. Data extraction focused on general information about the register, barriers to ascertainment, and strategies to support ascertainment and high data quality.

**Results:**

52 registers were identified, 20 completed the survey and 19 included in the study (CP registers, n = 10, NZ health registers, n = 9). Web scan data were included for the other 32 registers (CP registers, n = 21, NZ health registers, n = 11). Indigenous health equity was identified in the visions and aims of only two health registers. Ethnicity data collection was identified in nearly three quarters of survey respondents and a limited number of organizational websites. Over half of survey respondents described system, health provider/service, or workforce barriers to ascertainment. Strategies were categorized into collaboration, health provider/service, workforce, and systems-levels. Indigenous-specific strategies were limited and focused on personal behaviour and access to registration.

**Conclusions:**

CP and other health registers can have a significant role in identifying and addressing Indigenous health inequities. However, this is not currently an overt priority for many registers in this study and few registers describe ascertainment and data quality strategies specific to Indigenous peoples. Significant opportunity exists for health registers to be accountable and to implement approaches to support Indigenous health equity, address structural determinants of inequities, and achieve health gain for all.

## Background

Health registries play an important role in the systematic and structured monitoring and evaluation of health conditions. Established health registers have been shown to positively impact quality of care, health care policy and research [1–4] with evidence of optimized patient outcomes apparent in multiple clinical conditions, including cardiology, stroke, cancer, and cerebral palsy (CP) [5–8]. The European and Australian CP registers are two examples of how population-based data collection has been instrumental in identifying CP risk factors, aetiology, and prognosis, leading to the development of standardized care pathways and implementation of prevention-based strategies [3, 9–11]. Recent reports of a decline in the rates of CP (to 1.4 / 1000 live births) from both Australia and Europe [12, 13] are reflective of a combination of improvements in the monitoring of outcomes and preventative and early management [7].

However, improved outcomes are not universal. The prevalence of CP remains high in low-middle income countries (CP prevalence of 3.4/1000 live births in Bangladesh) [14], for Indigenous children, and those experiencing socioeconomic disadvantage [15, 16]. Globally, Indigenous health inequities are common across many health conditions and markers of health [17]. Determinants of ethnic health inequities, though complex and multifactorial, are driven by structural determinants such as unequal treatment by societal institutions (i.e., political, legal, economic and cultural), racism, and privilege [18]. For Indigenous peoples colonization is a common and ongoing trauma that systematically alienates populations from land, erodes Indigenous ways of knowing, being and doing, and produces systemic health inequities [19].

Complete, consistent, and high-quality health data is a strategic resource necessary to support Indigenous health and wellbeing [20], a right inherent to Indigenous peoples and reinforced in the United Nations Declaration on the Rights of Indigenous Peoples (UNDRIP) [21]. The use of health registries, containing complete and high-quality Indigenous datasets, are an integral means to both identify and monitor the health status of Indigenous children [22]. Established in 2015, the New Zealand Cerebral Palsy Register (NZCPR) is a pediatric-focused, confidential, standardized data collection that aims to increase understanding of the needs of people with CP in Aotearoa New Zealand (NZ). Current ascertainment for the NZCPR is approximately half of the expected CP pediatric population, with ethnicity profiles close to NZ population proportions (24% aged 0–21 years identifying as Māori (the Indigenous peoples of NZ)) (*unpublished findings from NZCPR*). However, given that some CP risk factors are disproportionately high for Māori in NZ (i.e., higher prevalence of low birth weight [23] and birth prematurity [24]), it is likely that Māori children and young people with CP are currently underrepresented in the NZCPR. As a key step towards supporting Māori health equity for people with CP, the NZCPR and researchers from Te Kupenga Hauora Māori, University of Auckland, are identifying approaches to ensure sustainable and comprehensive ascertainment of Māori with CP on the register, and the collection of high-quality data.

The aims of this study were to use environmental scanning methods consistent with Kaupapa Māori theory and practice to identify perceived and existing barriers for the ascertainment of Māori/Indigenous peoples on international CP registers and NZ health registers, and to collate strategies identified and used to support comprehensive ascertainment and achievement of high-quality data for Māori/Indigenous peoples. Although originating in the business sector as a method of identifying and assessing internal and external elements of an organization, environmental scanning is increasingly being utilised in government sectors and public health as a planning and quality improvement tool [25]. Outcomes of this work will provide CP and other health registers with the opportunity to identify and implement actions to support Indigenous health equity.

## Methods

### Research design

Kaupapa Māori theory, the theoretical framework for this research, is derived from Te Ao Māori (the Māori world) and supports critical, transformational, and empowering research that is ‘by’, ‘with’, and ‘for’ Māori [26–28]. Kaupapa Māori recognises the legitimacy and validity of Māori and Māori ways of doing, and the ongoing struggle for autonomy [29]. Within the context of this study, the principal investigator is Māori; co-investigators are Māori, Shona, and non-Māori non-Indigenous. ‘Ngā Poutama Whetū’ (NPW), translated to ‘stairway to the stars’, is a Kaupapa Māori narrative review framework [30] that was adapted to meet the aims and aspirations of the study. Each step and adaptations from the original NPW framework are described below. This study was also reported in accordance with the CONSIDER statement, used to strengthen the reporting of research involving Indigenous peoples [31]. Study criteria, data collection and data analysis are described in the following sections, demonstrating the application of the NPW framework to environmental scanning research methods.

### Kaupapa: collective aims and aspirations for Māori

This first step established study parameters that aim to produce research that is safe, ethical, and supportive of health gain for Indigenous people with lived experience of CP. This study focused on ascertainment of Indigenous peoples (i.e., the process of finding and recruiting Indigenous people on to a register) and Indigenous data quality in health registers. In alignment with the Kaupapa of this study, data quality refers to accuracy, completeness, reliability, relevance, and timeliness – an adaptation of the six dimensions of data quality introduced by Kerr, Norris and Stockdale [32]. There is no internationally agreed upon formal definition of Indigenous peoples, reflecting unique and distinctive Indigenous cultures, languages, and systems. Within the context of this study, Indigenous peoples are characterised by the United Nations “working definition”. This definition emphasizes self-determination as Indigenous, the fundamental importance of ancestral land and territory to collective physical and cultural survival, and “an experience of subjugation, marginalization, dispossession, exclusion or discrimination because of their different cultures, ways of life or modes of production than the dominant model” [33] (p.7). Ethics approval was granted by the Auckland Health Research Ethics Committee (Ref. AH21927) for the survey component of this study.

### Tino Rangatiratanga: self-determination

This step reinforces the autonomy of researchers to ensure alignment with the Kaupapa throughout the study. Tino rangatiratanga was demonstrated when identifying health registers, undertaking data extraction and the application of inclusion and exclusion criteria. For inclusion in the study registers were either i) a CP register based in a country with Indigenous populations, or ii) a health condition/disease register based in NZ. A list of CP registers was collated, and then Indigenous population confirmed using the UN working definition defined in the *Kaupapa* step. Health registers were defined as standardized datasets collecting systematic and structured data relevant to a health condition, therefore, other registers, such as organ donation and immunisation registers, were excluded.

### Kia piki ake i ngā raruraru o te kainga: socioeconomic mediation

This step, moved forward from step five of the original model, recognises people in contexts and maintains focus on structural determinants of health. Health registers were recognised as valid and legitimate sources of knowledge and data obtained through an online survey and a scan of organizational websites.

The survey was developed using Qualtrics© with questions pertaining to three key areas: (i) general information about the register including ethnicity data; (ii) barriers to ascertainment; and (iii) strategies to support ascertainment and data quality processes. NZ registers were also asked if they were interested in joining a network to support Māori health equity. Recruitment was purposive; the research team identified a total of 52 health registers (31 CP registers from countries with Indigenous populations and 21 NZ health registers). A register representative with access to the necessary information, often a research officer, was invited to complete the survey (February—March 2021). Periodic reminders were sent and the survey left open for a generous length of time to support participation and minimize selection bias.

A scan of register websites (from NZ health registers and international CP registers not responding to the survey) was then performed (March 2021). The aim of the website scan was to extract the same data as collected within the web-based survey, with the exception of barriers to ascertainment, utilizing publicly available information, policies, and procedures.

### Ako: culturally preferred pedagogies and reciprocity

This step involved organising information and appraising alignment of data as ‘by’, ‘with’ or ‘for’ Māori. Although obtaining data ‘by’ and ‘with’ Māori would be preferred, this was unlikely to align with the current structure of most health registers. Thus, data comprised of information that is ‘for’ Māori and Indigenous health gain including identifying systems and structures that inhibit or promote this. Consequently, survey and web scan data that was deemed to be deficit framing of Indigenous peoples was excluded. Deficit framing was defined as the identification of internal deficiencies as the cause of disparities [34], focusing on Māori/Indigenous culture or peoples as the problem [35]. The deliberate exclusion of deficit framing is consistent with a structural approach and prevents perpetuation of individual responsibility explanations and solutions that may, although often inadvertently, contribute towards Indigenous health inequities in health registers.

### Taonga tuku iho: treasures to pass on

This step is a continuation of organizing information and appraising alignment with the Kaupapa. The survey was pretested by NZCPR research officers to evaluate its functionality, sensibility and alignment with study aims. Inclusion and exclusion criteria were applied as described previously to identify registers and collect data. Our protocol and a final draft of the publication were peer-reviewed by a senior Māori researcher external to the team.

### Whānau: inter-relatedness with others

Whānau seeks to analyse data and synthesise data into themes. This research employed inductive thematic analysis to analyse data. An inductive approach to thematic analysis is data-driven; assuming that the identified themes are strongly linked to the data itself [36]. Within the NPW framework, the analytical phase can be broken down into whānau (extended family), hapū (sub-tribe) and iwi (tribe) [30]. From this perspective, the codes (whānau) are interconnected with others to form categories (hapū) and synthesised into themes (iwi). Data were extracted from the survey and web scan under five predetermined variables: (i) register characteristics, (ii) register vision and aims, (iii) ethnicity data collection, (iv) barriers to ascertainment (survey only), and (v) strategies to ascertainment and data quality. Data were coded by one researcher and reviewed and confirmed by two others.

### Kaupapa: collective aims and aspirations for Māori

This final step returns to the Kaupapa of the research through expression of the findings and dissemination. Findings will be reported or presented to the NZCPR governance group, survey respondents, clinicians, and Māori with lived experience of CP. In addition, findings have influenced NZCPR actions and strategic direction.

## Results

### Register characteristics

Of the 52 health registers invited to participate, 38% (n = 20) completed the survey and 19 were included in the study (international CP registers, n = 10, NZ health registers, n = 9). In the process of data extraction, one respondent was excluded prior to analysis as it was determined that the register did not meet the study inclusion criteria as a health register. Of the 10 international CP registers, respondents came from Asia (n = 1), North America (n = 1), Oceania (n = 5) and Europe (n = 3). Three of the 9 NZ health registers were joint NZ and Australia registers. Registers from the survey group were established from 1948 to 2017. Three pieces of data were excluded from coding and analysis as they identified barriers pertaining to Indigenous peoples’ behaviours and beliefs rather than systems or structures.

The web scan consisted of 32 health registers (international CP registers, n = 21, NZ health registers, n = 11). The international CP registers in this group came from Asia (n = 1), North America (n = 1), Oceania (n = 2) and Europe (n = 17). The year the registers were established ranged from 1959 to 2020 (for the sixteen registers where the date of establishment was identifiable on their websites).

### Visions and aims of registers

All 19 survey respondents provided either or both visions and aims for their health register, whilst visions and aims were only located for 15 of the 32 health registers included in the web scan (n = 5 CP registers, n = 10 NZ registers). The visions and aims were categorized into five distinct categories (Table 1). No survey respondents identified Indigenous health equity in vision and aims. Two health registers in the web scan specified equity-specific visions and aims, prioritizing the identification of Indigenous specific issues and data.

**Table 1:** Summary table of health register vision/aim categories from survey and web scan data

Category	Survey	Web scan
Involvement in activities	Research, knowledge generation, collaboration, planning services, creating clinical tools (n = 9)	Research, knowledge generation, collaboration, planning, responding to queries, surveillance, informing future care, informing resource needs, prevention programmes (n = 9)
Improve / optimise	Incidence, quality of care and healthcare-related outcomes, healthcare delivery / services, health outcomes, data quality, management of healthcare / health condition, participation, quality of life (n = 8)	Incidence, quality of care, access to the register and clinical services, outcomes, care pathways, health and wellbeing, health professional awareness of health condition, treatment (n = 7)
Descriptive epidemiology	Prevalence, incidence, outcome, predictors of outcome, mortality, causal pathways, access to care / treatment (n = 10)	Prevalence, causal pathways, management, severity, mortality / morbidity, condition impacts / effects (n = 4)
Being/ becoming a high-quality register	Complete data, providing “equal treatment”, being sustainable, having a well-coordinated team, aligning with best practice (n = 7)	Complete data, “equal treatment”, consistent coding, sustainable funding, research / initiatives (n = 4)
Monitoring	Data quality, quality of care, performance of healthcare / treatment / management, performance of health service delivery (n = 7)	Quality of care, clinically high-risk families (n = 2)

### Ethnicity data collection within registers

Ethnicity data collection was reported by 15 respondents in the survey and 14 were included for analysis (one respondent collected nationality and was excluded from ethnicity analysis). Nearly all respondents (n = 13) indicated ethnicity data collection from the time established. Of the 14 registers collecting ethnicity data, all identified at least one data source, with 64% (n = 9) using more than one source for a combination approach. One respondent described an ethnicity algorithm protocol collecting data from three sources. Ethnicity data was obtained from a health record (86%, n = 12), self-reported/identified by person or caregiver (57%, n = 8), and from data linkage to national/regional datasets (36%, n = 5).

Six respondents collected Indigenous status, three collected total ethnicity (i.e., collecting all ethnic groups), two collected “primary” ethnicity (i.e., collecting one ethnic group from a pre-determined list), and one collected limited ethnicity (i.e., collecting and storing up to three ethnicities). Two survey respondents provided insufficient information on their ethnicity data collection method to be categorised.

From the web scan, the collection of ethnicity was identified in eight of the 32 registers, with identifiable data sources for seven. One register was connected to multiple ethnicity data collection sources. Each of the remaining six registers appeared to use one source. Ethnicity data sources included hospital records (n = 2), self-identification (n = 4), healthcare provider (n = 1), registration forms (n = 3), data linkage (n = 1), and unknown (n = 1). Based on the identified information, ethnicity data collection was categorised into limited ethnicity (n = 2), parental ethnicity (i.e., the ethnicity of the parent of the person on the register) (n = 3), and unknown (n = 4).

### Barriers and strategies

Over half (53%, n = 10) of survey respondents perceived there to be systems/structures that act as barriers to ascertaining Māori/Indigenous peoples, however, only two described targeted strategies/measures for Māori/Indigenous peoples. An additional two more registers described strategies/measures for ascertainment that did not report barriers. Five respondents (26%) had strategies in place for supporting data quality for Māori/Indigenous peoples. Barriers to ascertainment, ascertainment strategies and data quality strategies from survey respondents were categorized, and themes presented in Tables 2, 3.

**Table 2:** Summary table of themes from survey respondents’ perceived barriers to ascertain Indigenous peoples, and strategies to ascertain and support data quality of Indigenous peoples. Blank cells indicate no reported barriers/strategies

**Theme**	**Perceived barriers**	**Ascertainment strategies**	**Data quality strategies**
Collaboration		Involving IP	Involving IP
		• As advisors	• Indigenous advisory group
Systems	Ethnicity data quality		Ethnicity data systems and processes
	• Incomplete		• Standard ethnicity data protocol
	• Inaccurate		• Data linkage
	• Misclassified		• Indigenous status validation checks
	• Not collected		
Health provider/service	Insufficient information about register	Finding people	Reporting
		• Establishing access to rural/remote locations	• Audit
	Rurality of IP^#^	• Family friendly approach	Activities specific to Indigenous data quality
	Indigenous health equity not prioritised	Centering equity	• Indigenous-specific research
		• Equity groups	• Quality improvement initiatives for IP
		• Equity-focused meetings	• Multiple data collections
			Post data collection processes
			• Quality assurance
Work force	Workforce capacity	Workforce capacity	
	Lack of IP	Region-based coordinator^ (also Finding People)	
	Regional workforce shortage		

IP = Indigenous peoples

^#^“Rurality of IP” refers to respondent perceptions that Indigenous peoples are predominantly a “rural population base”

*^*“Region-based coordinator” refers to placing “a coordinator in [a specific] region recognising a large number of [Indigenous] families with [the health condition] were located there”

**Table 3:** Summary table of themes from survey respondents’ strategies to ascertain and support data quality of the target population. Blank spaces indicate no reported strategies

Theme	Ascertainment strategies	Data quality strategies
Collaboration	Data	Data
	• Compare and share	• Use other data sources
	Clinical and community services	Health professionals
	• Building relationships and awareness	• To confirm diagnostic information
		Family
		• Consent to contact families to confirm/update data
Systems		Standard protocols / systems
		• Electronic registration system
		• Standardized data collection system
		• Data management plans
		Data collection processes
		• Double data entry
		• Data linkage
		Post data collection processes
		• Identify outliers
		• Quality assurance
		• Built in validations and completion checks within data entry system
		• Primary source verification
		• Data comparison
		• Receive feedback
		Update systems
		• National quality improvement projects
		• Update services
		• Semi-automated data import
Health provider/service		
	Understanding and valuing the register	Reporting
	• Champions	• Compare data
	• Ethics approval	• Quality audits
	• Reporting	• Validation audits
	• Newsletters	• Data quality indicators
	• Infographics	• Monitor change/trends
	• Annual reports	Post data collection processes
		• Reminders
	Registration process	• Feedback to stakeholders
	• Opt off/out consent	
	• Dedicated staff	
	• Registration via health professionals, other service providers, online and self-registration	
	Data collection	
	• Electronic data collection for ease of access	
	• Assisted data entry	
Work force		Workforce development
		• Annual workshops
		• Data team training and support for data collectors
		• Coordinator as advisor to data collectors

Table 4 outlines the themes, categories and codes established from the web scan across the pre-determined variables of ascertainment and data quality strategies for the total population. Selected whānau codes are expanded below. Within the ‘collaboration’ theme, “Analytical and research support” refers to the use of an external organization for data-driven and evidence-based analytical and research support. “Use other data sets” refers to the use of hospital admission data to support data quality. Under ‘Systems’, “Data progress tracker” refers to the use of an online tracker that measures progress on data transfer by each clinical provider.An Indigenous specific strategy was identified in only one website. “Guided by Treaty of Waitangi principles” was identified by one health register as an ascertainment strategy. The Treaty of Waitangi, signed in 1840, establishes the relationship between Māori and the British Crown. Specific detail on application of principles was not provided.

**Table 4:** Summary table of themes from web scan data strategies to ascertain and support data quality of the target population. Blank spaces indicate no reported strategies.

Themes	Ascertainment strategies	Data quality strategies
Collaboration		Networks
		• Analytical and research support
Systems		Data management systems
		• Data monitoring system
		• Data back up
		Data collection processes
		• Use other data sets
		• NHI* look up
		Post data collection processes
		• Data comparison
		• Data linkage
		• Completeness checks
		Update and monitor systems
		• System that learns and evolves
		• Data progress tracker
Health provider/service	Finding people	Reporting
	• Health service notification	• Regular reporting
	• Health professional referral	• Audit
	• Other datasets	• Clinic review
	Registration process	• Annual data review
	• Consent to contact	• Patient Reported Outcome Measures
	• In person registration	Data collection processes
		• Repeat data collection
		• Identify trends/change
		Quality improvement activities
		• Quality improvement programs
		• Targeted quality improvement projects

*NHI = National Health Index, is a unique identifier that is assigned to every person who uses health and disability support services in New Zealand

### Māori health equity network for New Zealand registers

Of the nine NZ survey respondents, all completed the Māori health equity network question. Of those, six were interested in joining a network of health registers with the purpose of advancing Māori health equity, all indicating knowledge sharing as a potential benefit. The ability to collaborate (i.e., aligning data dictionaries, comparing challenges, access to Māori health networks) was also perceived by some to be a potential benefit and one register indicated that such a network would support equity of outcomes.

### The use of the Ngā Poutama Whetū

NPW has been successfully applied to environmental scanning methods, which speaks to the versatility of this framework. The framework ensured the research findings benefitted Māori/Indigenous peoples by being strengths based, transformational and excluding deficit framing. Inclusion of deficit approaches, even if critiqued, risks legitimization and perpetuation of ascertainment and data quality strategies that are damaging to Indigenous peoples. Deficit approaches perpetuate negative stereotypes and explanations by focusing on individual responsibility or community behaviours, overlooking structural factors and the impacts of colonization.

## Discussion

To date, published peer-reviewed literature to support evidence-informed Indigenous-specific ascertainment and data quality policy is scarce [37]. The present study contributes towards this research gap by collating and critiquing register perceptions of barriers and currently implemented strategies.

Of note, although numerous strategies were described, strategies were predominantly focused on the total register population and not specific to Indigenous peoples. Furthermore, the relative absence of Indigenous health equity in the study registers’ vision and aims was salient. Egalitarian framings of equality were more apparent, with study registers describing aspirations to provide the same level of care and “equal treatment” to all “regardless of ethnicity”. Although well intentioned, equal treatment and color blind intentions inhibit, rather than promote, health equity through framing of ethnic inequities in terms of socioeconomic status, cultural difference, and individual behaviour [38]. Structural determinants, such as institutional racism – “differential access to goods, services, and opportunities of society by race” [39], are omitted and the racial status quo upheld. Egalitarian concerns with equality (sameness) over equity (fairness) support determinants of Indigenous health inequities to be abstruse within colonial health register systems, structures and dominion [40]. Subsequently, Indigenous health equity requires a process of active decolonization [41, 42], the critical examination of and challenge to colonial hegemony [43].

The inherent right to Indigenous health and equity is affirmed by UNDRIP, a declaration to which NZ and 147 other states are signatories. Health registers play an important part in upholding these rights by ensuring accurate identification and monitoring of health condition-related inequities for Indigenous peoples, including severity of disease, distribution of risk factors, and access to health services. Limitations in health register ethnicity data are concerning and represent a significant barrier to identifying Indigenous peoples and inequities. Of concern, more than a quarter of the registers included within this study were not collecting ethnicity and, therefore, were not able to identify Indigenous peoples within their register. In addition, study findings indicate that the quality of ethnicity data was sub-optimal for many registers. In NZ, standardized protocols guide the health and disability sector in best practice for ethnicity data collection, requiring self-identification, standardized question and responses, and recording up to six responses [44]. In contrast, ethnicity within the included registers is frequently captured from existing health records, infrequently self-identified, and often a single or limited number of ethnicities recorded, potentially misrepresenting self-identified ethnicity [45]. Our study observations are consistent with previous research outcomes, reporting inconsistent, irrelevant and poor quality health data related to Indigenous peoples [46]. There lies significant opportunity for all health registers, and indeed national and regional health systems, to align with best practice ethnicity data collection, storage, and analysis to support Indigenous health gain and equity. While some challenges around ethnicity data collection and reporting may require legislative change, inclusion of alternative data sources to increase identification of Indigenous populations may be warranted in specific situations, being mindful of Indigenous data sovereignty and ethnicity data quality of these sources.

A range of ascertainment and data quality strategies were described by the study registers; however, there are significant gaps in the breadth of strategies when a structural lens is applied. First, many barriers and strategies are orientated around personal behaviour and registration. In reality, registration as a personal behaviour is influenced by a multitude of complex factors outside of individual control [47], including socio-cultural conditions and discrimination from both within and outside of health systems. Second, rurality is used as an explanation for Indigenous barriers to healthcare access, yet in the NZ context and likely many others, this explanation is inaccurate. Although the proportion of Māori living rurally in NZ is greater than more urban areas, the vast majority of Māori in NZ live in urban settings [48]. The implication of personal behaviour and rurality framing is that strategies addressing the foundational drivers of Indigenous health inequities – colonization, racism, privilege and unequal treatment by institutions [18, 19] – are left unaddressed [47]. Third, Indigenous peoples were not recognised as having a role beyond being ‘advisors.’ For quality Indigenous data to be obtained, data must be relevant to Indigenous peoples and, therefore, aligned with Indigenous worldviews and preferences [46, 49]. This requires meaningful involvement of Indigenous peoples and worldviews, particularly those with lived experience of health conditions, across all levels of health register governance and decision making [50, 51].

**Fig. 1:**
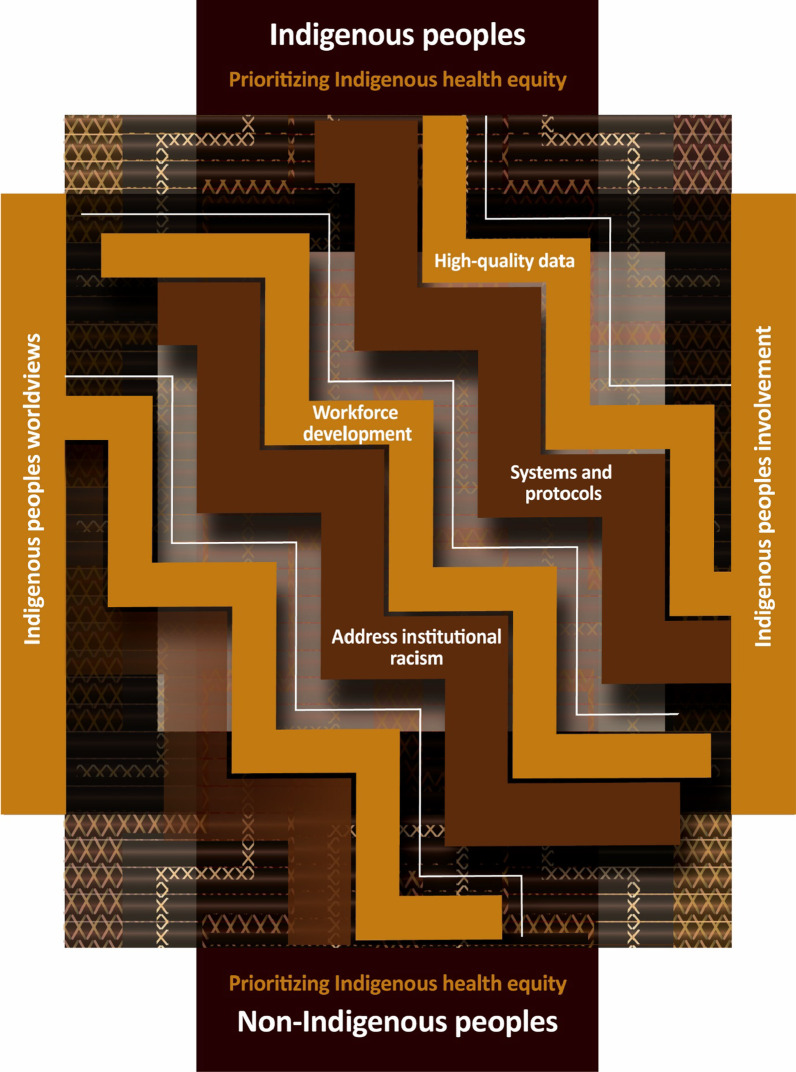
A preliminary framework for embedding Indigenous health equity in health registers

It is recognized that countries have unique and distinct populations and constitutional journeys and, therefore, have diverse barriers to the expression of Indigenous self-determination through an independent Indigenous-led health register. Integrating Indigenous worldviews and approaches provides the opportunity to strengthen existing health registers, benefiting both Indigenous and non-Indigenous populations. Outcomes of this work have identified several opportunities for health registers, such as the NZCPR, to centre Indigenous health. Critical to this approach, is that Indigenous self-determination is upheld. A tukutuku panel of the poutama pattern has been used as a preliminary framework to illustrate how health registers might centre Indigenous health equity (Fig. 1). A tukutuku panel is the ornamental lattice work used between carvings around walls of Māori meeting houses (wharenui). Poutama is the stepped pattern, symbolising genealogies and levels of learning and achievement [52]. The structure of the tukutuku panel is comprised of a gold rod on either side with multiple black horizontal rods between them. Indigenous worldview and Indigenous involvement represent the gold rods and the prioritisation of Indigenous health equity the black cross bars. This structure provides a solid and safe frame for weaving, a health register environment supportive of Indigenous health equity.

The weaving of stepped stitches occurs alongside other steps with no obvious end, indicating that the steps do not occur in isolation and are ongoing. The inclusion of high-quality data for Indigenous people, standardised evidence-based systems and protocols, workforce development activities to support capacity and capability to support Indigenous health equity, and actions to address institutional racism and discrimination are represented by the stepped stitches. Each activity is related to the other and is an ongoing process throughout the duration of a register. Finally, it takes two people to weave a tukutuku – one in front and one behind. This represents the roles of Indigenous and non-Indigenous peoples. The tukutuku cannot be created without working in a supportive and collaborative way and this requires clear understanding of roles, relationships and governance that supports self-determination for Indigenous peoples.

This study has numerous strengths. Utilizing a Kaupapa Māori approach has ensured Māori worldviews and Indigenous health equity have been centred. The use of both a survey and web scan has resulted in the collection and analysis of data from a broad range of CP registers and NZ health registers. A NZ community of practice has been established after more than half of NZ health register respondents expressed interest in an informal network to support advancement of Māori health equity actions, providing a platform to support sharing of knowledge and collaboration. Limitations to this study are also recognised. Whilst the survey provided concise information to fit within pre-determined variables, less data were obtained through the web scan. This is unsurprising given the specificity of information being sought, which may not be considered relevant or appropriate to position on a register website. It is possible that further Indigenous specific strategies and measures may be undertaken by health registers that were not identified by our methodology. Furthermore, it is necessary to observe the limitations of data being ‘for’ rather than ‘by’ or ‘with’ Māori. The framing of barriers is from the perspective of respondents and health registers, not of Indigenous peoples themselves. It is recognised that understanding of barriers should come from the individuals affected themselves, an undertaking that was outside the scope of this study, but of significant valuable to future research.

## Conclusions

Cerebral palsy and other health registers play a key role in the health sector, offering valuable insight for health providers and for people with lived experience of health conditions. Despite the continued need to address the stark inequities that exist between Indigenous and non-Indigenous peoples’ health outcomes, very few registers in this study appear to overtly prioritise Indigenous health equity, and some, though well-meaning, may inadvertently perpetuate and increase inequities by espousing “equal treatment”. Significant opportunity exists to identify and implement approaches and strategies that address structural determinants and support equitable ascertainment and data quality for Indigenous peoples. This includes meaningful involvement of Indigenous peoples and worldviews, a workforce with sufficient capacity and capability to ascertain and collect high-quality data from Indigenous peoples, and activities to address institutional racism. Achieving Indigenous health equity requires understanding of and overt commitment to health equity, resulting in transformation supportive of high-quality health registers and health gain for all.

## Data Availability

The datasets analysed during the current study are available from the corresponding author on reasonable request.

## Abbreviations

CP: Cerebral palsy
IP: Indigenous peoples
NPW: Ngā Poutama Whetū
NZ: Aotearoa New Zealand
UNDRIP: United Nations Declaration on the Rights of Indigenous Peoples
